# Irritability, Poor Feeding and Respiratory Alkalosis in Newborns: Think about Metabolic Emergencies. A Brief Summary of Hyperammonemia Management

**DOI:** 10.3390/pediatric12030019

**Published:** 2020-10-25

**Authors:** Stefano Del Re, Aurélie Empain, Alfredo Vicinanza, Ovidiu Balasel, Anne-Britt Johansson, Jean-Philippe Stalens, Corinne De Laet

**Affiliations:** 1 Neonatal Intensive Care Unit, Hôpital Universitaire des Enfants Reine Fabiola, Université Libre de Bruxelles, 1020 Brussels, Belgium; ovidiu.balasel@huderf.be (O.B.); anne-britt.johansson@huderf.be (A.-B.J.); 2 Department of Nutrition and Metabolism, Hôpital Universitaire des Enfants Reine Fabiola, Université Libre de Bruxelles, 1020 Brussels, Belgium; aurelie.empain@huderf.be (A.E.); corinne.delaet@huderf.be (C.D.L.); 3 Pediatric Intensive Care Unit, Hôpital Universitaire des Enfants Reine Fabiola, Université Libre de Bruxelles, 1020 Brussels, Belgium; alfredo.vicinanza@huderf.be; 4 Neonatal Non-Intensive Care Unit, Centre Hospitalier de Wallonie Picarde (Site Union), 7500 Tournai, Belgium; jean-philippe.stalens@chwapi.be

**Keywords:** hyperammonemia, respiratory alkalosis, urea cycle disorder, newborn, continuous venovenous hemodiafiltration

## Abstract

The urea cycle is a series of metabolic reactions that convert ammonia into urea in order to eliminate it from the body. Urea cycle disorders are characterized by hyperammonemia, which can cause irreversible damages in central nervous system. We report a series of three newborns presenting irritability, poor feeding and tachypnea. Their first gas analysis revealed respiratory alkalosis. Hyperammonemia was confirmed, and three different enzymatic blocks in the urea cycle were diagnosed. Immediate treatment consisted in the removal of ammonia by reduction of the catabolic state, dietary adjustments, use of nitrogen scavenging agents and ultimately hemodiafiltration. Hyperammonemia is a medical emergency whose treatment should not be delayed. This report aims to highlight the importance of suspecting urea cycle disorders in newborns with aspecific signs of hyperammonemia and respiratory alkalosis, and to sum up the broad lines of hyperammonemia management.

## 1. Introduction

The urea cycle is a metabolic pathway involved in nitrogen detoxification and arginine synthesis [1]. It requires six enzymes and two transporters [2]. Any total or partial enzyme deficiency or transport defect in this pathway leads to a urea cycle disorder (UCD), resulting in acute hyperammonemia except for the arginase deficiency [3].

Hyperammonemia is neurotoxic and can cause irreversible damages in the central nervous system; it has to be considered as a medical emergency [4]. The initial symptoms of hyperammonemia are hyperventilation with irritability evolving to coma [1]. Hyperventilation in UCDs is a common early finding which causes respiratory alkalosis. That is thought to result from astrocyte swelling responsible of cerebral edema caused by the accumulation of ammonia, glutamine and other metabolites when osmoregulation is insufficient [5]. Increasing cerebral edema may result in progressive encephalopathy with hypoventilation and respiratory arrest. 

Urea cycle disorders have autosomal recessive inheritance pattern except for ornithine transcarbamylase (OTC) deficiency, which is X-linked [6]. The estimated combined incidence of these disorders is 1/55,000 live births in the USA and in Europe [7].

The initial presentation can occur at any age; approximately half of all patients are newborns developing symptoms within the first 12 to 48 h after birth. Different factors as catabolism (acute infection, fever, surgery, trauma, fasting) or increased protein intake may trigger the hyperammonemic crisis [8]. No specific clinical signs could help distinguish the different types of urea cycle defect, except for hyperargininemia (or arginase deficiency), whose clinical manifestations may progressively take hold in affected subjects, such as progressive spastic diplegia (or quadriplegia) as well as ataxia and developmental delay around the age of two to four years [1]. 

The diagnosis of UCD can be made either on clinical basis or by neonatal screening in some countries. Newborn screening allows an early diagnosis and intervention even before symptoms appear. Screening programs are essentially based on the Wilson and Jungner criteria [9] and target illnesses with important health issue whose early diagnosis and treatment can lead to a direct improvement in the patient’s quality of life. However, variations in the criteria for neonatal screening have emerged as a result of technological progress (molecular biology, mass spectrometry), medical advances and even public demand. Thus, the list of diseases that can now be detected in the neonatal period is becoming technically very important and should be constantly updated. Therefore, the number and nature of the screened diseases vary enormously by country [10]. In Belgium, both the Dutch speaking and the French speaking communities use the Guthrie test for their neonatal screening programs. The list of the diseases to screen is decided by each community, according to their own legal criteria. In the French speaking community, argininosuccinate synthase (ASS) and argininosuccinate lyase (ASL) deficiencies can be detected but are not routinely requested. Carbamoyl-phosphate synthase 1 (CPS1) and OTC deficiencies are more difficult to detect because their metabolites are technically difficult to quantify [10].

We report three cases of newborns with irritability, tachypnea and feeding problems. Their initial blood gas analysis showed respiratory alkalosis, thus suggesting hyperammonemia. This observation allowed a rapid diagnostic orientation with adequate therapeutic management.

## 2. Case Reports

The three reported patients were admitted to neonatal or pediatric intensive care units between April and October 2017. 

Their gestational ages ranged from 36 to 41 weeks. Pregnancy was properly monitored and uneventful for all of them. Birth weights were normal for gestational age, except for case 3 who was dysmature (Table 1). No consanguinity was reported among the parents of the three patients. They did not require any cardiopulmonary resuscitation in delivery room. They were admitted to the neonatal or pediatric intensive care units between the first and the seventh day of life owing to similar symptoms as poor feeding, lethargy, irritability and sleepiness. Respiratory distress and hyperventilation were remarkable as well (Table 1).

Their infectious laboratory workups were negative. The first blood gas analysis revealed respiratory alkalosis for all of them, which led us to measure ammonemia. Initial plasma ammonia levels were high between 527 and 788 µmol/L (normal range: 48–110 µmol/L) (Table 1). As metabolic disorders were suspected, urea cycle intermediates were especially investigated. 

**Figure 1:**
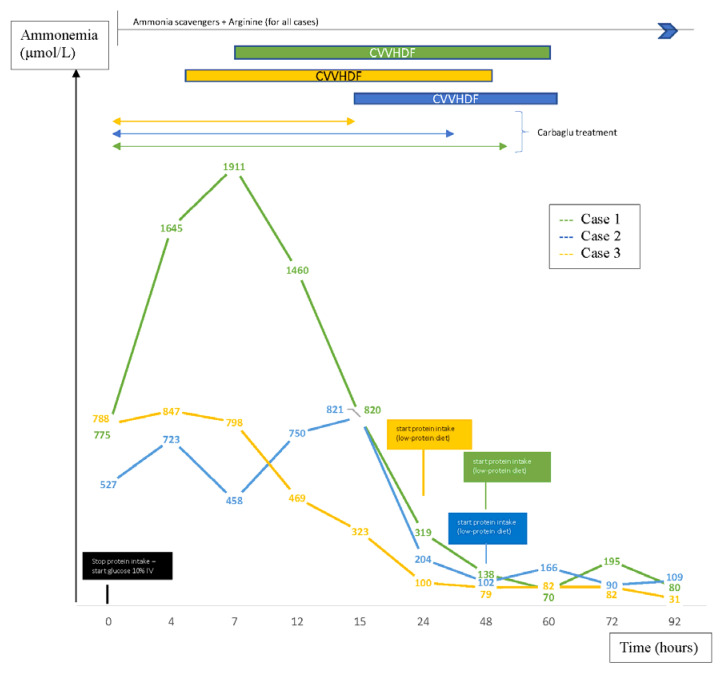
Evolution of ammonemia.

The immediate first therapeutic line for all patients consisted in invasive ventilation support because of neurologic impairment, glucose infusion (10 mg/kg/min) after stopping protein intake, administration of ammonia scavengers (intravenous sodium benzoate) and arginine. Protein-free therapy was instituted rapidly to prevent a rebound of plasma ammonia levels. Parenteral nutrition containing lipids and glucose was assured to optimize caloric intake and to prevent catabolic state. Proteins and essential amino acids (EAA) were reintroduced after 24 to 48 h. As since enteral feeding was tolerated (between two and eight days after admission to intensive care units), the patients received a low-protein diet adapted to plasma ammonia levels and plasma amino acids. Despite first-line therapy, hyperammonemia persisted in each case and blood gases progressed to acidosis for the two boys. Therefore, continuous venovenous hemodiafiltration (CVVHDF) was rapidly initiated for all of them, thus permitting a significant decrease in plasma ammonia levels (Figure 1).

Biochemical investigations including hepatic enzymes, glycemia, lactatemia, plasma amino acids, urinary orotic acid, urinary ketone bodies and urinary organic acids were performed to find the enzyme deficiency. Plasma glutamine was increased in all patients. Three different UCDs were identified: the ornithine transcarbamylase (OTC) deficiency, the carbamoyl-phosphate synthetase (CPS) deficiency and the argininosuccinic acid synthetase (ASS) deficiency.

Carglumic acid, generally used for the treatment of hyperammonemia in patients with N-acetylglutamate synthase deficiency [1,11] was started empirically along with the first therapeutic line. Due to its inefficiency in the management of hyperammonemia in our patients, and after receiving the results of the first biological tests (12 to nearly 48 h after starting dialysis), which excluded a deficiency in N-acetylglutamate synthase, this treatment was, therefore, stopped (Figure 1).

Electroencephalography, performed shortly after admission to intensive care units, was pathological in all of them, showing an epileptic activity or asynchronous slow waves with clinical signs of encephalopathy. Thus, anticomitial therapy was administered to manage seizures. Electrophysiological, as well as neurologic signs improved in all patients, thus correlating with the ammonia plasma level drop. Cranial ultrasounds were non-contributory in all cases. Nevertheless, one to two weeks after the onset of symptoms, every patient had abnormal cerebral magnetic resonance showing the presence of ischemic lesions in sparse spots pattern or abnormalities in white matter without cerebral edema.

Genetic analysis confirmed these three diagnosis. Different methods were used including the parallel mass sequencing method (on a panel of 3989 genes associated with metabolic diseases). The mutations found in the ASS 1, CPS 1 and OTC 1 genes confirm the three diagnosis (Table 1). These variants, already described, are associated with the observed phenotypes. A genetic analysis was also requested from the parents of our patients: case 1’s mother was found to be a carrier of the mutation; for case 2, both parents are found to be carriers of the mutation; the parents of case 3, unfortunately, were not able to perform genetic analysis. 

Their treatment at discharge was the same: low-protein and high-energy diet, sodium benzoate, L-citrulline and arginine. 

At 14 months of age, the psychomotor development for case 3 was normal. Her movements were fluid and well-coordinated. Case 2 exhibited mild axial hypotonia at the same age; despite this, his movements were fluid and well-coordinated as well. Nevertheless, he required Bobath physiotherapy support. Their growth parameters were within normal limits for age. Unlike the other ones, case 1 had more severe neurologic sequelae with overall development delay, axial hypotonia and persistent seizures. 

Because of the poor quality of life, the constraints due to the diet with recurrent metabolic decompensations and hospitalizations, cases 1 and 2 underwent a liver transplant at 10 and 16 months of age, respectively. 

## 3. Discussion

This report aims to highlight the importance of non-specific signs and especially respiratory alkalosis in newborns as a clue of suspicion for urea cycle disorders. Hence, this might lead clinicians to measure ammonemia in such neonatal cases. 

Moreover, urea cycle disorders are not frequently encountered in the routine practice of pediatricians and neonatologists. Consequently, most physicians have relatively little experience in the management of hyperammonemia. As hyperammonemic crisis could cause severe neurologic sequelae, this should be considered as a medical emergency and first-line treatment should not be delayed. Immediate treatment is important in term of prognosis. Therefore, starting from the description of three cases, another purpose of this work is to take a look at the acute management of hyperammonemia in newborns. 

Hyperventilation in UCDs is a common early finding which causes respiratory alkalosis. As noted in two of our cases, respiratory alkalosis can progress to acidosis, mostly if UCDs are diagnosed and treated late [1,5]. A blood gas analysis is, therefore, useful to guide the diagnosis of this type of metabolic disorders. Respiratory alkalosis (high serum bicarbonate and high arterial pH) with normal anion gap and blood glucose in a newborn should prompt immediate plasma ammonia measurement, as hyperammonemia is initially present in 50% of acute urea cycle disorders [1]. 

Hyperammonemia is defined as a plasma ammonia level greater than 110 µmol/L for neonates born at term. However, hyperammonemia is present in UCDs mostly at higher values (>200 µmol/L) [4]. If the ammonia plasma value is between 110 and 200 µmol/L, a control is recommended. For premature infants, upper limit values of ammonemia may be higher (between 110 and 200 µmol/L). An idiopathic disorder known as transient hyperammonemia of the newborn (THAN) can occasionally be present in preterm newborns as well, characterized by a normal blood glutamine level and not always symptomatic [1]. If plasma ammonia is elevated, further basic laboratory and metabolic investigations (determination of plasma amino acids, plasma acylcarnitines, urinary organic acids and orotic acid) might be immediately carried out without delaying specific treatment [1].

**Figure 2:**
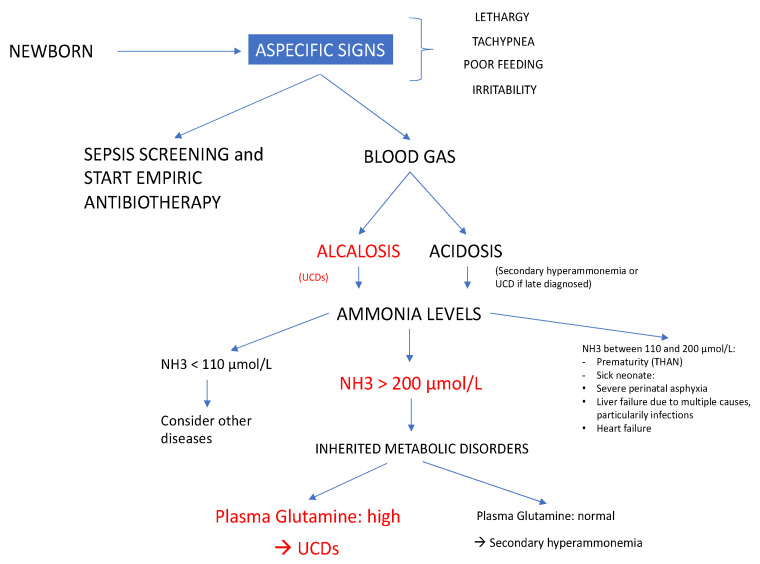
Diagnostic algorithm for newborns presenting aspecific clinical signs of hyperammonemia. NH3: ammonia; UCDs: urea cycle disorders; THAN: Transient hyperammonemia of the newborn.

The initial symptoms of UCDs are very aspecific in newborns: tachypnea, lethargy, irritability, poor feeding and sleepiness. The most common misdiagnosis is neonatal sepsis [12]. In Figure 2 we propose a diagnostic algorithm useful for newborns presenting aspecific clinical signs of hyperammonemia.

Besides UCDs, other causes of hyperammonemia should be considered as well. Hyperammonemia in neonates is either primary, due to an enzymatic block in the urea cycle, or secondary, due to organic acid disorders, fatty acid oxidation defects, congenital sepsis or severe hepatic insufficiency [13]. The plasma level of glutamine and urea cycle intermediates (citrulline and arginine) are helpful to distinguish between primary and secondary hyperammonemia [1]. The presence of urinary ketone bodies, hypoglycemia, hyperlactatemia, hypertransaminasemia, elevated serum creatine phosphokinase and uric acid may help address diagnosis towards metabolic deficits [1]. All of these biological values were normal in our three patients. Urea cycle disorders were suspected rapidly because of the respiratory alkalosis, the presence of elevated plasma glutamine level and disturbed plasma values of urea cycle intermediates.

Our cases manifested typical severe neonatal forms of urea cycle disorders as they presented, in the very first days of life severe hyperammonemia, encephalopathy and seizures.

Lately, patients’ management for UCDs is becoming more uniform in Europe according to the literature, mostly on the basis of the recently updated Häberle guidelines [1], which we apply as well.

The management of UCDs combines dietary and pharmacological therapy. The first line therapy is to provide a protein-free caloric intake, better based on intravenous 10% glucose at 10 mg/kg/min (to prevent endogenous protein catabolism) and should be started as soon as hyperammonemia is detected.

Moreover, ammonia scavengers are the main drugs useful to bypass the urea cycle, thus contributing to reduce plasma ammonia level. All of our patients were treated with sodium benzoate. The choice of ammonia scavengers varies by countries: sodium phenylbutyrate is more used in North America while sodium benzoate is more common throughout Europe [14]. However, both are effective choices, and are recommended when plasma ammonia levels are between 150 and 250 μmol/L. Furthermore, arginine and/or citrulline supplementation could maximize ammonia elimination from the body through the urea cycle [1]. Although carnitine is still part of this acute treatment in Europe (particularly in Spain), our patients did not receive this molecule because it has been proven to have little benefits in UCDs and it is, therefore, not recommended anymore by the latest European guidelines [1,14].

Continuous venovenous hemodiafiltration (CVVHDF) should be initiated urgently in patients unresponsive to dietary and pharmacological treatments, perhaps systematically when plasma ammonia levels rise above 500 μmol/L [1]. Despite the first therapeutic line, increased plasma ammonia levels were still observed in all our patients, which led us to start CVVHDF. As soon as severe hyperammonemia is detected, the patient should be immediately transferred to a center where hemodiafiltration is available.

Proteins should be ideally reintroduced when ammonia plasma level falls below 100 µmol/L as in cases 2 and 3, or at the latest after 48 h of a protein-free caloric intake, as in case 1, in order to prevent catabolism, avoid negative nitrogen balance and prevent a rebound of plasma ammonia levels [1].

Dietary treatment for UCDs varies significantly among European Inherited Metabolic Diseases centers. In these patients, it has been recommended to administer the 20–30% (up to 50% in ARG1 deficiency) of the entire protein intake as EAA in order to maintain growth and metabolic control [1]. Nevertheless, in a study by Adam S et al. including 464 patients with UCDs from several European countries, only 30% of the cases in United Kingdom received this EAA supplementation and in the rest of the Europe these rates average 38%, with notable differences among countries (100% in Sweden, 67% in Portugal, 64% in Germany, 38% in Belgium, 29% in Italy and 24% in France) [15]. In the USA, EAA are rather recommended for patients with UCDs [16]. Our three newborns had a protein restricted diet and received EAA as suggested by the European guidelines [1].

Early diagnosis and management are the key to minimize neurologic sequelae. Hyperammonemia might generate irreversible injuries to the development of the central nervous system: cortical atrophy, ventricular enlargement and demyelination leading to cognitive impairment, seizures, mental retardation and spastic quadriparesis [5,6]. Seizures are present in almost 50% of neonates diagnosed with hyperammonemia [17] and were observed in all our newborns. The prognosis and neurodevelopmental outcome should be considered in the therapeutic assessment as well [1]. Neurologic outcome is very poor in presence of intracranial hypertension and/or if plasma ammonia peaked at above 1000 μmol/L, although the impact of this level on prognosis depends on the duration of hyperammonemia [1]. The case 1 reached the highest plasma levels with a peak above 1000 μmol/L during almost 12 h and developed later serious psychomotor development issues. Damages in central nervous system are reported to be irreversible when peak plasma ammonia levels rise above 480 µmol/L already [1,5]. Nevertheless, case 3 had a normal psychomotor development at 14 months of age even though she had the highest initial plasma ammonia level at diagnosis, but of short duration and with rapid improvement (normalization of ammonemia 12 h after starting treatment). It has been described that neurologic sequelae depend on the length of the hyperammonemic coma as well: they usually occur when the exposure to hyperammonemia is longer than two days [18]. 

In Europe, there is a wide geographic variation in the number of screened metabolic diseases. In general, even at equal levels of evidence, due to ethical and legal issues and societal implications in countries with different health systems, decisions on which diseases to include in neonatal screening programs remain heterogeneous. Italy is the European country where the highest number of inborn errors of metabolism (IEM) are tested in neonatal screening programs (including, among others, type 1 and type 2 citrullinemia, argininosuccinic aciduria and hyperargininemia for the UCDs). In Italy, this number averages 27 IEMs (with more than 40 in Tuscany) compared to 10 IEMs tested in Belgium [19]. However, some European countries (such as France, Germany, Austria, Switzerland or Denmark) consider that most pathologies affecting the urea cycle are not eligible for inclusion in the group of diseases to be screened at birth for several reasons: high number of false positives, lack of consensus on the individual benefits of early intervention, low number of patients described in the literature, early onset of symptoms before results of test are known and lack of evidence on the effectiveness of screening [20]. In our institution, UCDs are not usually screened at birth neither.

Genetic testing are performed in order to confirm diagnosis. Mutation analysis is the method of choice for definitive diagnosis of UCDs, to help genetic counseling and in some instances to indicate the prognosis [1].

## 4. Conclusions

Timely diagnosis of hyperammonemia requires a high index of suspicion. Plasma ammonia should be measured in all newborns with unexplained symptoms such as poor feeding, irritability, lethargy, particularly in presence of tachypnea. The presence of respiratory alkalosis is an early sign that will guide the diagnosis for urea cycle disorders. As delay in treatment may result in progressive neurologic injury, or even death, a correct diagnosis should be established as soon as possible. The appropriate conservatory management is critical. The access to the CVVHDF should be anticipated and granted if unsatisfactory response to first-line therapy.

As UCDs represent an important health problem because of irreversible sequelae if misdiagnosed and untreated, a single rapid and valid method of screening to help diagnosis might be desirable all over Europe.

## Figures and Tables

**Table 1:** Personal information, symptoms, diagnostic laboratory data and diagnosis of the cases.

	Case 1	Case 2	Case 3
Gestational age (weeks)	41 + 1/7	39	36
Birth weight (grams)	3260	3830	2035
Gender	Boy	Boy	Girl
Admission to intensive care unit (days of life)	2	1	7
Symptoms at admission	Sleepiness; Hypotonia Irritability; Tachypnea	Irritability and poor feeding	Sleepiness; Lethargy; Irritability; Tachypnea
Initial blood gas analysis (capillary)	Respiratory alkalosis (pH 7.60; pCO2 22.3 mmHg; HCO3 21.5 mmol/L)	Respiratory alkalosis (pH 7.52; pCO2 24 mmHg; HCO3 19.5 mmol/L)	Respiratory alkalosis (pH 7.55; pCO2 26 mmHg; HCO3 22 mmol/L)
Initial ammonia plasma level (µmol/L)	775	527	845
Blood amino acids	Low plasma levels of citrulline, argininosuccinic acid and arginine; High plasma level of glutamine	Low plasma levels of citrulline and arginine; High plasma level of glutamine	High plasma level of citrulline; High plasma level of glutamine
Urinary orotic acid	High	Normal	High
Diagnosis	Ornithine transcarbamylase (OTC) deficiency	Carbamoyl-phosphate synthetase (CPS) deficiency	Argininosuccinic acid synthetase (ASS) deficiency
Genetic analysis	Mutation in c996G > A (p.Trp332) of the OTC gene in a haemizygote state	Missense mutation c.4142C > T (pLeu1381Ser) in exon 35 of the CPS 1 gene in a heterozygous state	Mutation in variants c.773 + 49C > T and c.1168G > A (pGly390Arg) of the ASS1 gene in a heterozygous state
Length of stay in intensive care unit	28	24	8
